# Supplementary material for: Cytokine-induced memory-like responses in endothelial cells link chronic inflammation to vascular disease risk
Source: Mol Omics. 2025 Oct 14;21(6):706–22. doi: 10.1039/d5mo00136f (PMC12519785; doi:10.1039/d5mo00136f)
Supplement: MO-021-D5MO00136F-s001 [file MO-021-D5MO00136F-s001.pdf]

This journal is © The Royal Society of Chemistry 2025

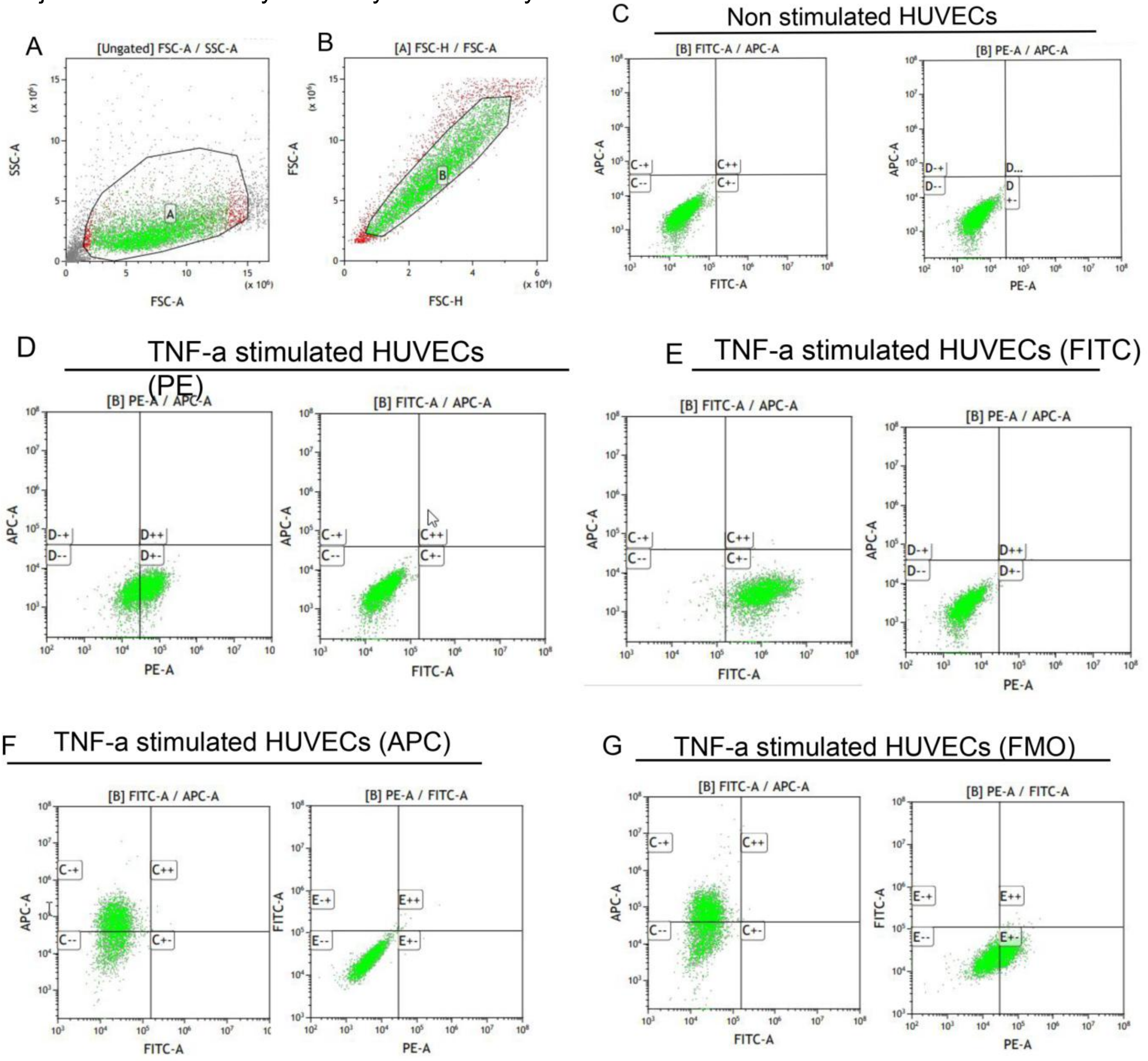

Figure S1: Gating strategy for flow cytometry analysis. Each dot or point on the plot is a representation of an individual cell passing through the laser. Intact cells were selected by gating an area illustrated by forward scatter (FSC-A) and side scatter (SSC-A) **(A)**. Single cells were selected based on the FSC-H and FSC-A **(B)**. Unstimulated cells, stained with IgG isotope controls were used for setting the gate for each florescent marker. Muti-color compensation was calibrated using activated HUVECs (TNF-α for 4 hours) **(C)**. E-selectin-positive cells (PE) were identified, while ICAM-1 (APC) and VCAM-1 (FITC) signals remained negative **(D)**, ICAM-1 positive cells (FITC) were identified, while E-selectin (PE) and VCAM-1 (APC) remained negative. **(E).** VCAM-1 positive cells (APC) were identified while E-selectin (PE) and ICAM (FITC) remained negative **(F)**. A fluorescence minus one (FMO) control was included, where all markers were stained, except FITC **(G).**

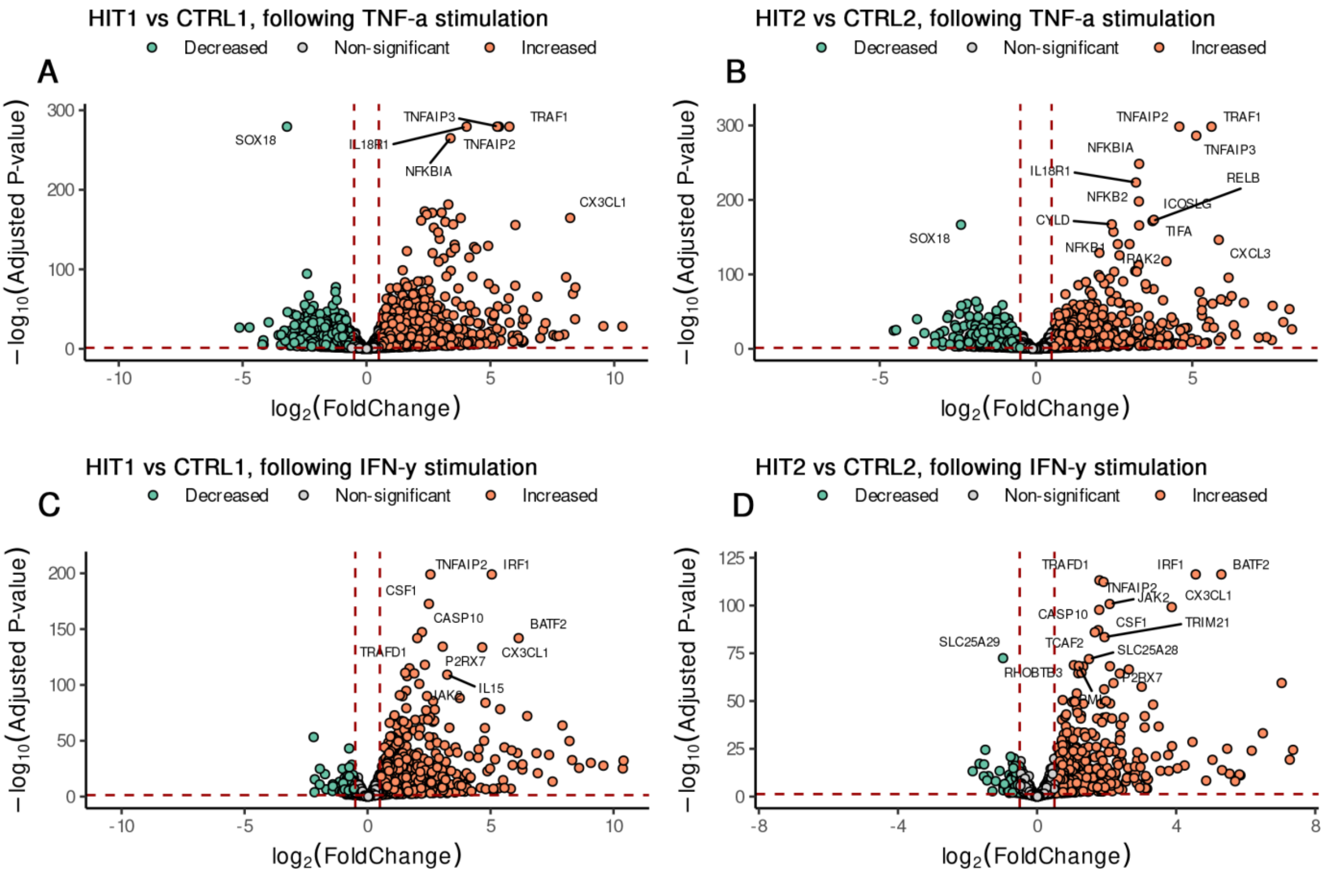

Figure S2: Volcano plots depicting transcriptional changes in ECs upon TNF-α and IFN-γ stimulation. Volcano plots showing DEGs identified in different conditions, HIT1 vs CTRL1 **(A)** and HIT2 vs CTRL2 **(B)** for TNF-α stimulation, and HIT1 vs CTRL1 **(C)** and HIT2 vs CTRL2 **(D)** for IFN-γ stimulation. Increased (orange) and decreased (green) DEGs are highlighted, with top 10 genes labeled. DEGs were filtered based on FDR adjusted p-values ≤ 0.05 and log2 fold change (log2FC ≥ 0.5) thresholds. The x-axis represents the $\log_2$ fold change, while the y-axis visualizes the -$\log_{10}$ transformed FDR-adjusted p-values.

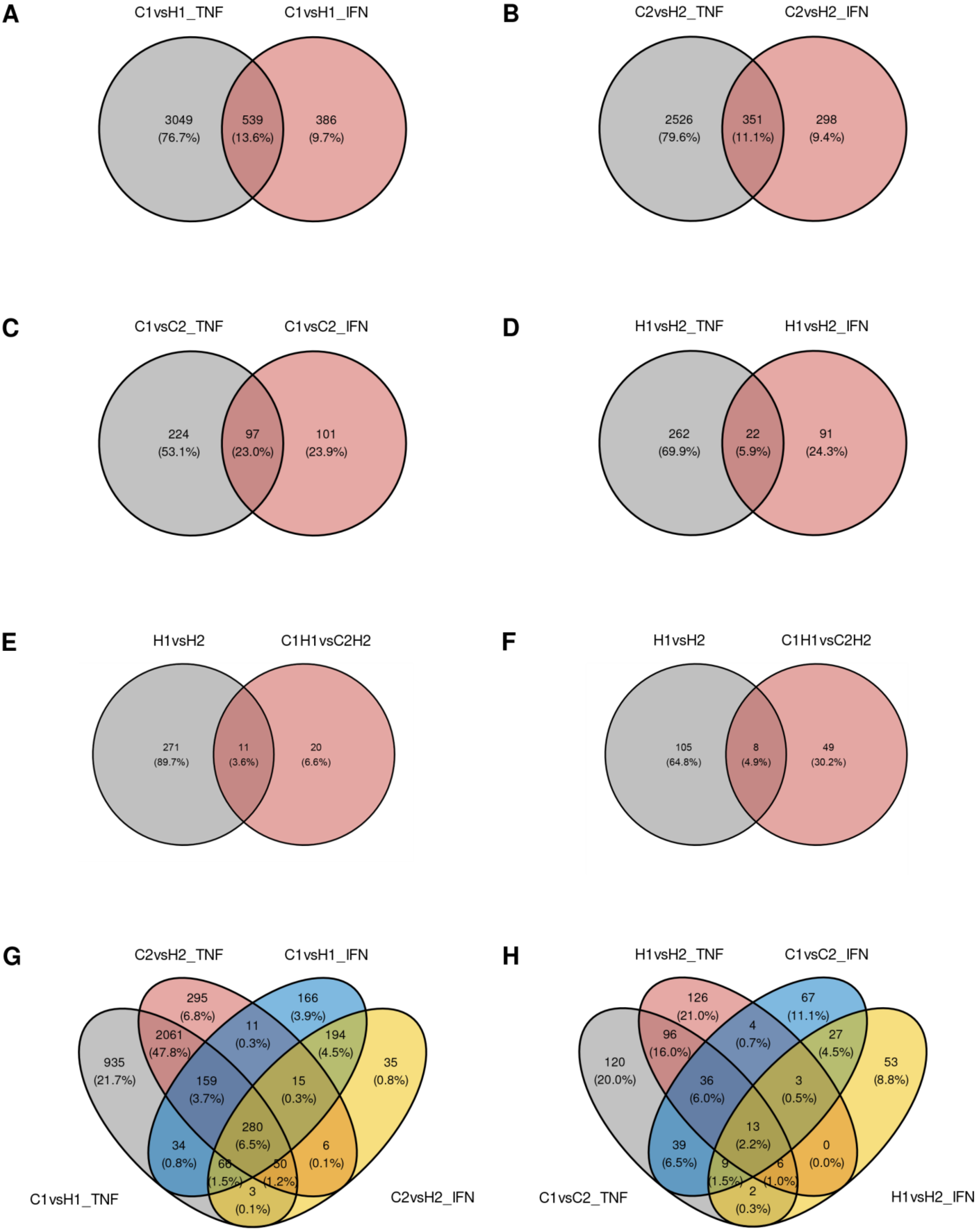

Figure S3: Overlapping genes between different stimulation conditions
Venn diagram visualizing the overlapping genes between the TNF-a and IFN-y stimulation after single stimulation (HIT1 vs CTRL1) **(A)** and repeated stimulation (HIT2 vs CTRL2) stimulation **(B)**. Whereas the overlapping genes between the control conditions (CTRL2 vs CTRL1) between TNF-a **(C)** and IFN-y **(D).** Overlapping genes between the stimulation (HIT2 vs HIT1) and (HIT2 – CTRL2 vs HIT1 – CTRL1) conditions are depicted for TNF-α **(E)** and IFN-y **(F).** Lastly, we compared the first (HIT1 vs CTRL1) and second (HIT2 vs CTRL2) stimulation of both cytokines **(G),** and the control (CTRL2 vs CTRL1) and stimulated (HIT2 vs HIT1) conditions **(H).**

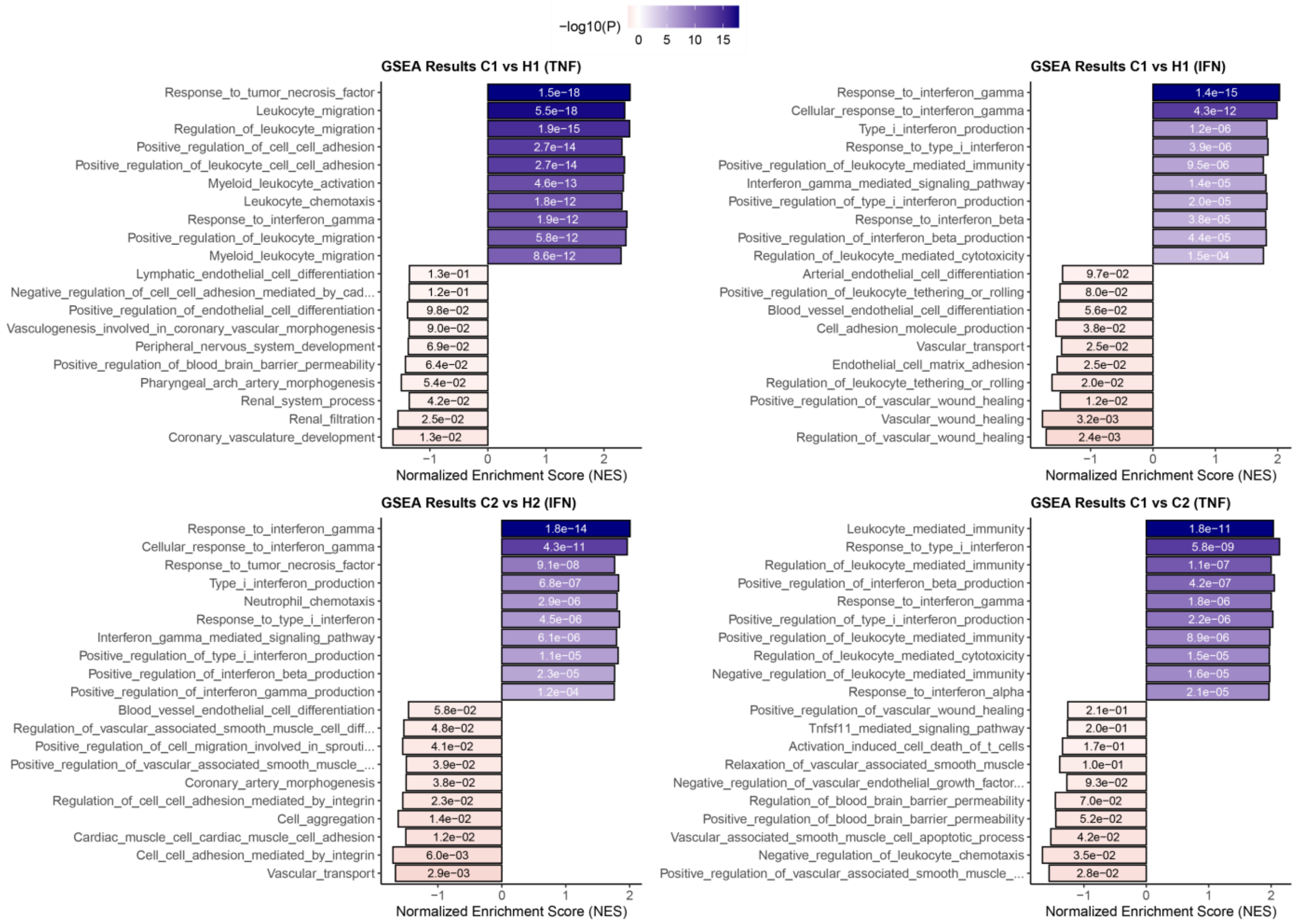

Figure S4: Enrichment of endothelial specific pathways across conditions. Gene set enrichment analysis (GSEA) was performed using curated endothelial specific gene sets derived from GO biological process. Results for the single-hit stimulation (HIT1 vs CTRL1) **(A)** and repeated expose (HIT2 vs CTRL2) **(B**) following TNF-α stimulation. Similarly, results of the single-hit stimulation (HIT1 vs CTRL1) **(C)** and repeated exposure (HIT2 vs CTRL2) (**D**) following IFN-γ stimulation. Blue bars indicate upregulated pathways, while red bars indicate downregulated pathways. The x-axis indicates the normalized enrichment score (NES), and the intensity of the colors reflects the –log10 FDR adjusted p-value

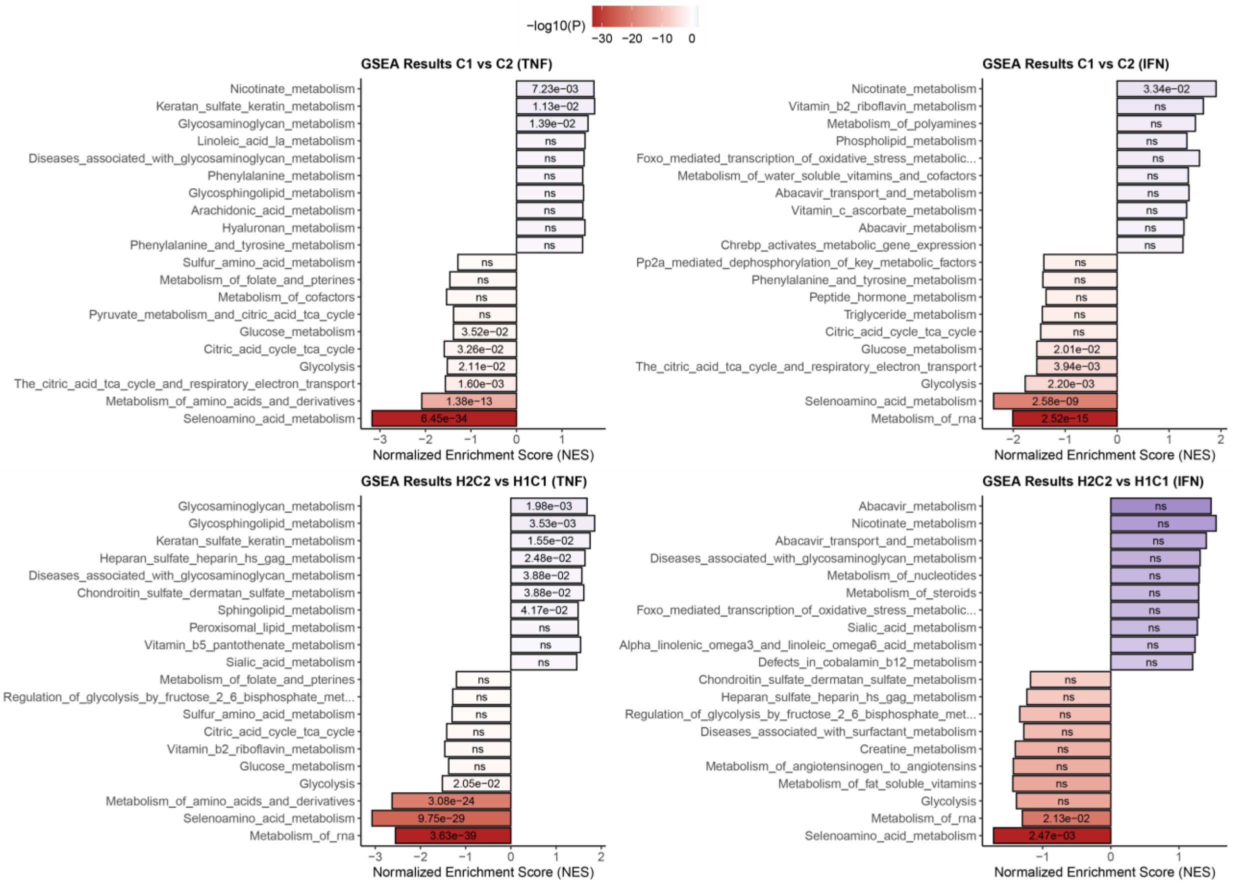

Figure S5: Enrichment of metabolic specific pathways across conditions. Gene set enrichment analysis (GSEA) was performed using metabolic specific gene sets derived from Reactome. Results for the control conditions (CTRL2 vs CTRL1) **(A)** and baseline-adjusted comparison(HIT2 - CTRL2 vs HIT1 – CTRL1) **(C)** following TNF-α stimulation. Similarly, results of the control stimulation (CTRL2 vs CTRL1) **(B)** and baseline adjusted (HIT2 - CTRL2 vs HIT1 – CTRL1) (**D**) following IFN-γ stimulation. Blue bars indicate upregulated pathways, while red bars indicate downregulated pathways. The x-axis indicates the normalized enrichment score (NES), and the intensity of the colors reflects the –log10 FDR adjusted p-value

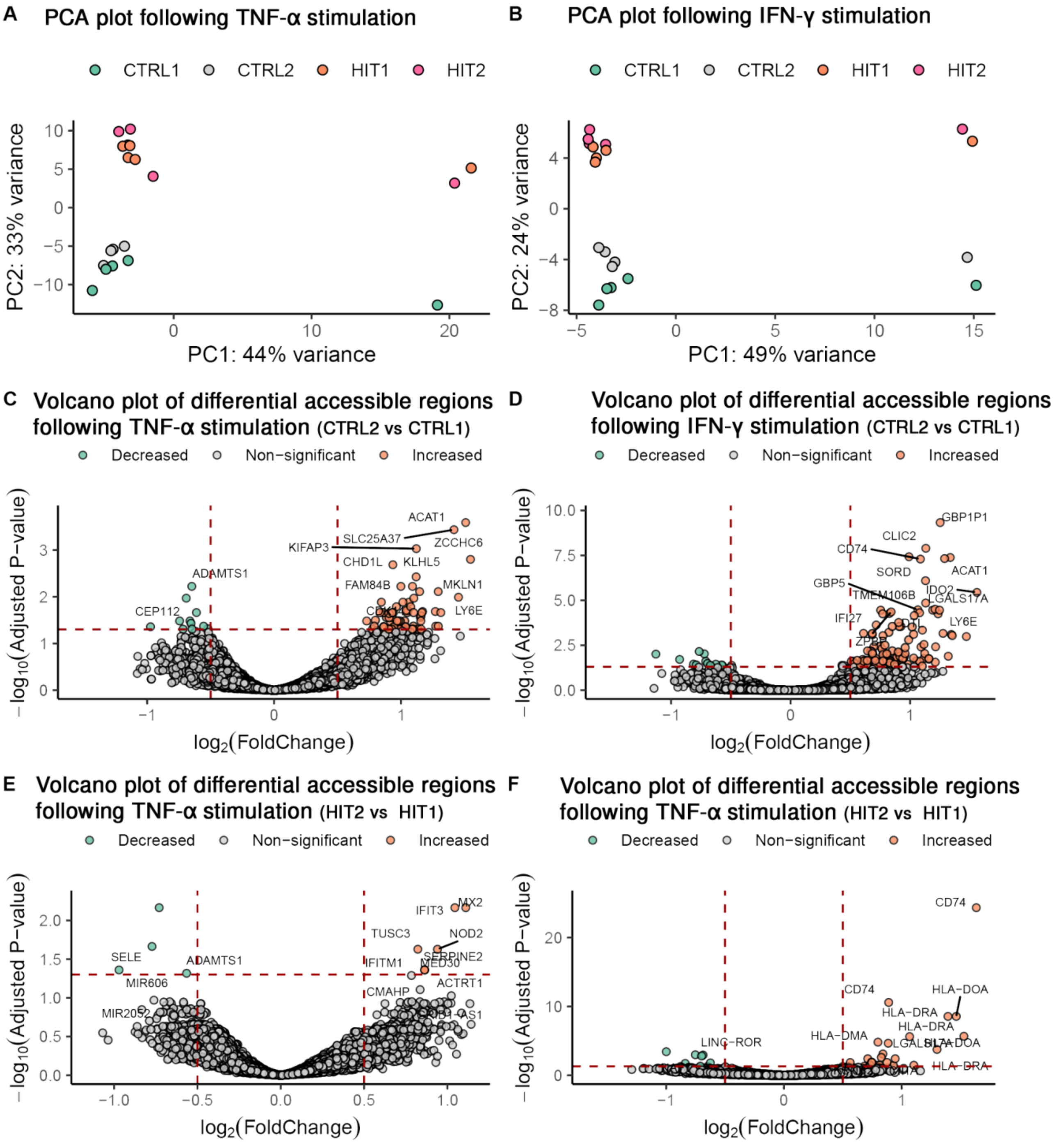

Figure S6: PCA and volcano plots depicting chromatin accessibility and transcriptional changes in ECs upon TNF-α and IFN-γ stimulation. PCA plots show the variance in chromatin accessibility across four conditions following TNF-α stimulation **(A)** and IFN-γ stimulation **(B).** Colors depict the different stimulation conditions. Volcano plots showing DORs identified in different conditions, CTRL2 vs CTRL1 for TNF-α **(B)** and IFN-γ **(C),** HIT2 vs HIT1 for TNF-α **(D)** and IFN-γ. Increased (orange) and decreased (green) DORs are highlighted, with top 10 DORs are labeled with gene names. DORs were filtered based on FDR adjusted p-values ≤ 0.05 and log2 fold change thresholds.

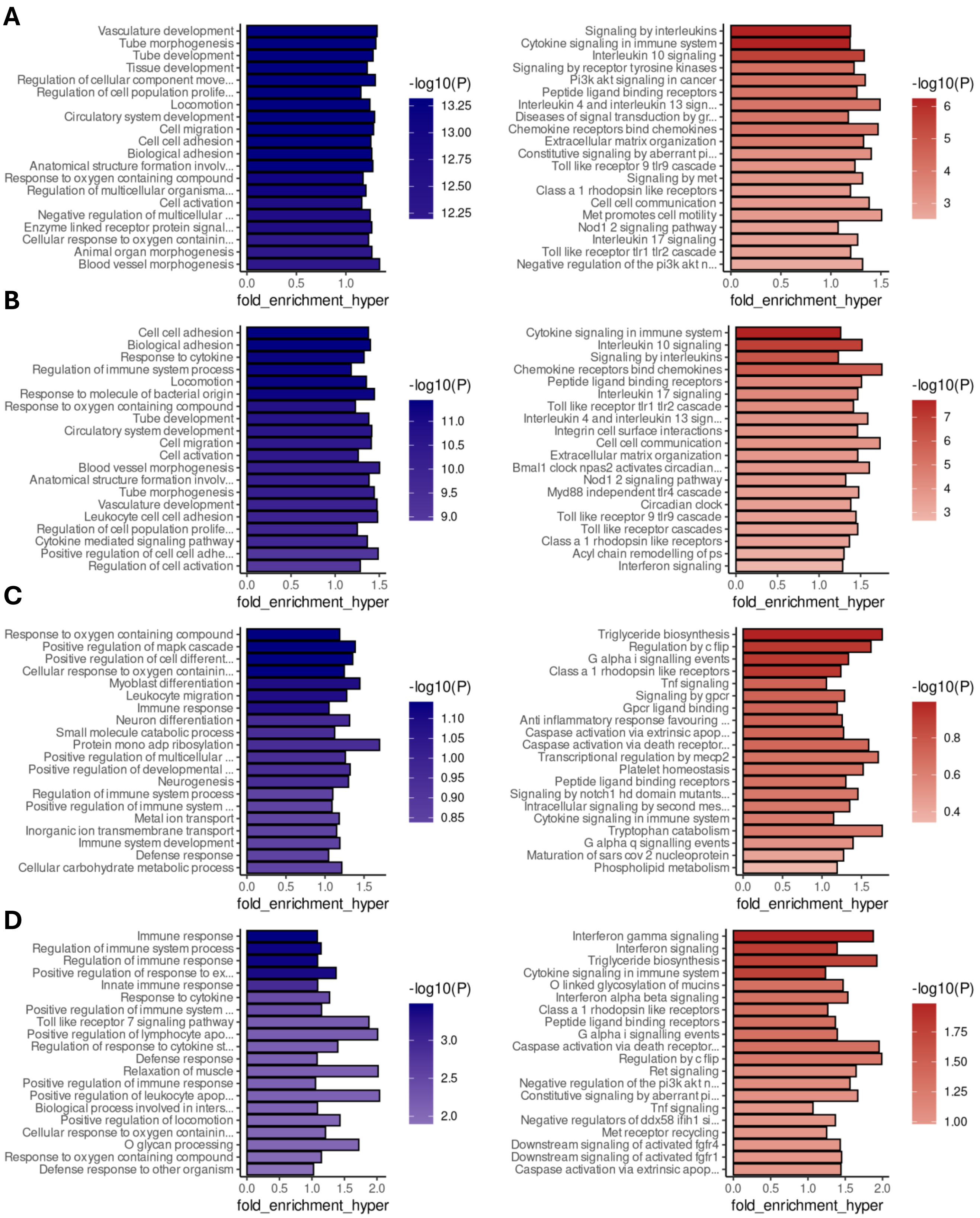

Figure S7: Enrichment results of DORs across conditions using both GO biological process and Reactome. Pathway enrichment results for the single-hit stimulation (HIT1 vs CTRL1) **(A)** and repeated expose (HIT2 vs CTRL2) (**B**) following TNF-a stimulation. Similarly, results of the single -hit stimulation (HIT1 vs CTRL1) **(C)** and repeated expose (HIT2 vs CTRL2) **(D)** following IFN-y stimulation. Blue bars represent results for GO biological process, while red bars represents Reactome pathways. The x-axis indicates the enrichment score, while the intensity of the colors represents the –log10 FDR adjusted p-value

A

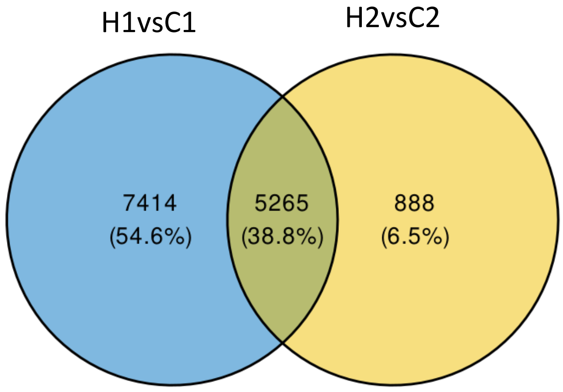

B

	H1C1	H2C2	Shared
Promotor-TSS	1999 (27%)	275 (31%)	1237 (23.5 %)
Exon	202 (5%)	52 (5%)	157 (3%)
Intron	2885 (39%)	317 (26%)	2159 (41%)
Intergenic	2119 (28%)	245 (27%)	1632 (31%)

C

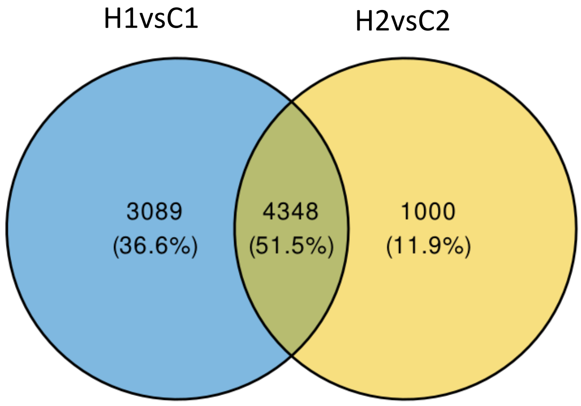

D

	H1C1	C2H2	Shared
Promotor-TSS	978 (31%)	261 (26%)	1051 (24%)
Exon	76 (5%)	50 (5%)	106 (5%)
Intron	1121 (36%)	391 (39%)	1838 (32%)
Intergenic	841 (28%)	298 (30%)	1242 (29%)

Figure S8: Overlap and genomic distribution of differentially open regions (DORs) upon single and repeated cytokine stimulation. Venn diagrams visualizing the overlapping DORs between the single stimulation (HIT1 vs CTRL1) and repeated stimulation (HIT2 vs CTRL2) for TNF-α **(A),** and IFN-γ **(C)**. Percentages represent the proportion of DORs unique to each condition or common between them. Distribution of different genomic regulatory regions are of DORs for TNF-α **(B)** and IFN-γ **(D).** The table provides the total number and the percentages of DORs in each category.

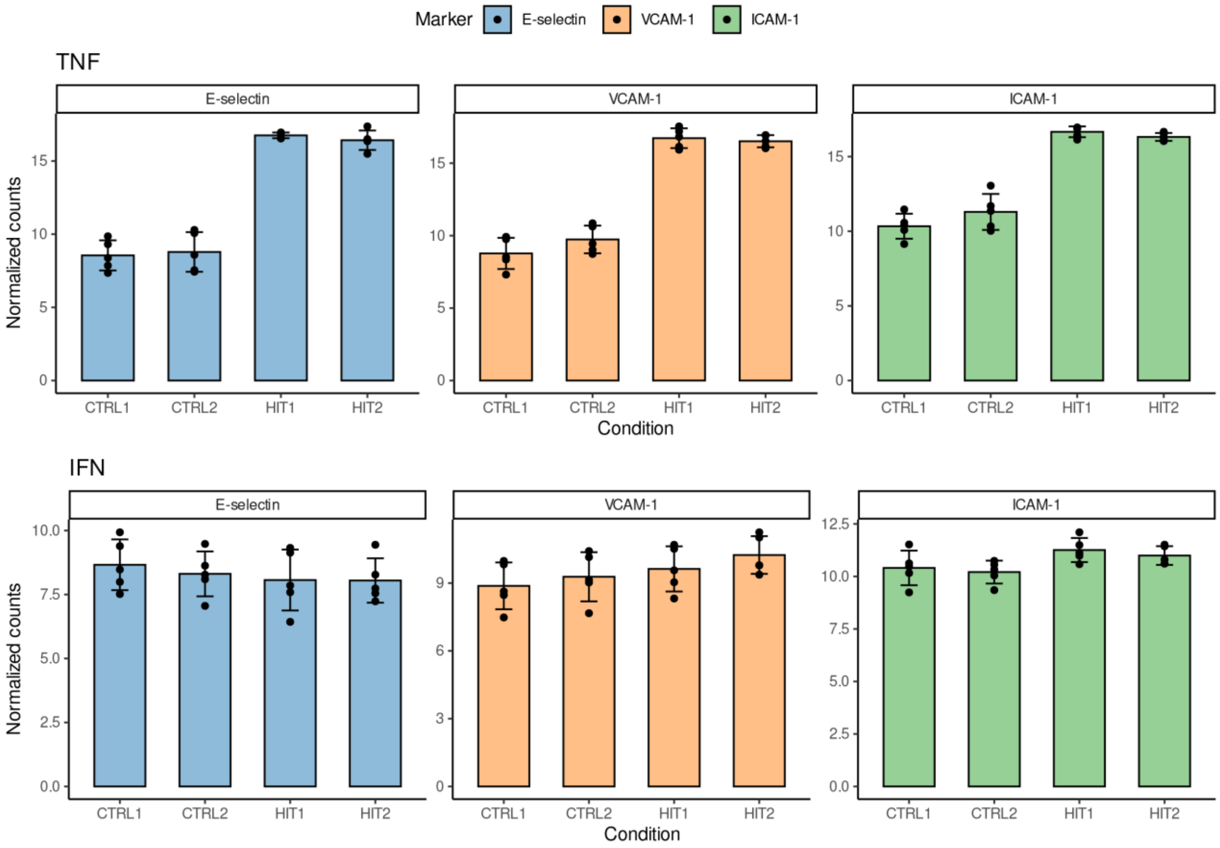

Figure S9: Transcriptional profiles of adhesin molecules
Bar plot representing the RNA-seq transcript levels of adhesion molecules E-selectin, VCAM-1, and ICAM-1 under different experimental conditions, following cytokine stimulations. Error bars indicate the standard error of the mean (SEM) derived from biological replicates (n = 5). The y-axis represents the normalized RNA-seq count data, while the x-axis represent different stimulation conditions.
